# Comment on “Prevalence and determinants of poor sleep quality among patients with diabetes mellitus”: addressing confounding and sampling limitations

**DOI:** 10.1097/MS9.0000000000004152

**Published:** 2025-10-17

**Authors:** Karan Chaman Lal, Ayesha Ubaid Ullah, Mishal Iqbal, Zainab Binte Tahir, Syeda Zainab Kazmi, MD Faisal Ahmed

**Affiliations:** a Department of Medicine, Liaquat University of Medical & Health Sciences, Jamshoro, Pakistan; ‎b Department of Medicine, Islamic International Medical College, Rawalpindi, Pakistan‎; c Department of Health Science and Informatics, Bangladesh Institute of Innovative Health Research, Mirpur, Dhaka-1216, Bangladesh

*Dear Editor,*

This study strictly adheres to the TITAN Guidelines and emphasizes ‎that other studies should follow this[1].

We read Muhammad Hamayal *et al*’s article[2] with great interest. This article is exceptionally well written, and ‎we ‎commend the authors for addressing poor sleep quality among patients with diabetes mellitus. However, ‎despite such ‎input, the authors have drawn a few drawbacks that limit the generalizability and reliability of the ‎study.‎

Firstly, in this study, the regression model tested broad comorbidities and uncontrolled diabetes‎, clearly shown in Table 5, which included only demographics like age, gender, BMI, income, ‎education, glycemic control, medications, etc. There is no inclusion of depression, anxiety, ‎obstructive sleep apnea (OSA), or neuropathy assessment. Even though the authors highlight ‎the importance of sleep determinants such as restless leg syndrome, peripheral ‎neuropathy associated with unbearable pain, and OSA in patients with diabetes mellitus, they still ‎left out these confounders in the model. Deepak Khandelwal *et al* clearly stated that multiple factors may ‎contribute to insomnia and that depression is one of the most important factors contributing to ‎poor sleep. Also, the prevalence of OSA in type-2 diabetes may be as high as 23% and the ‎prevalence of any sleep-disordered breathing as high as 58%. Hence, they proved OSA as a ‎well-established independent predictor of poor sleep quality and poor glycemic control[3], whilst the ‎present study did not adjust for it. Therefore, without assessing these central confounders, the ‎regression model becomes internally invalid and the reported associations are potentially biased.‎

Secondly, the author claims that metformin causes disturbed sleep, leading to nightmares, ‎disrupted sleep cycle, and even insomnia. However, this statement is in direct conflict with a ‎much larger set of experiments. An analysis of UK Biobank data from more than 11 000 patients ‎with type-2 diabetes showed no recognizable differences in the symptoms of insomnia or disturbed ‎sleep cycles between patients on metformin monotherapy and those on other antidiabetic ‎therapies. The study also observed that patients receiving non-metformin-based therapies ‎‎(primarily sulfonylureas) reported a 1.24 times higher risk of trouble initiating or maintaining sleep ‎than both the untreated and metformin-treated groups. Thus, it can be concluded that metformin ‎does not negatively impact sleep and may even be indirectly beneficial[4]. Consequently, high-quality data provide sufficient evidence to reject the author’s claim and regard metformin as ‎sleep-neutral. A more impartial statement would be to acknowledge that the evidence is ‎inconsistent and to recommend further long-term studies.‎

Additionally, this study showed significant associations between poor sleep quality and ‎socioeconomic variables such as low income and limited education, but these findings were ‎derived only from chi-square tests, which are univariate analyses and do not adjust for ‎confounders. Even though the authors included clinical risk factors such as age, comorbidities, ‎duration of diabetes, and glycemic control in their logistic regression but they did not include ‎income or education despite their univariate significance. This can result in a significant ‎drawback as socioeconomic status is known to influence sleep and diabetes outcomes; thus, not ‎including it can lower the validity of the study. However, Yi-Shu Liao *et al* included the level of education and socioeconomic status in their multivariate ‎regression models, thus emphasizing the independent effect of education on both diagnostic ‎complications and mortality risk in patients with diabetes. This highlights the limitations of the ‎study as well as demonstrates the importance of socioeconomic factors[5]. Therefore, ‎incorporating socioeconomic factors in multivariate models would guarantee more ‎accurate results for future studies.‎

Moreover, a major shortcoming of the study includes a heavily skewed age distribution. Out of 372 ‎individuals, nearly three-quarters (73.4%) were older than 45 years, while representation of ‎younger age groups was almost negligible. Merely 2 participants were in the 5- to 15-year age ‎group, and participants under 30 were scarcely represented. As a result, the study mainly ‎reflects older diabetic patients, making it hard to generalize the findings to younger adults, who ‎show different disease patterns and sleep disturbances. A Saudi study by Abdulaziz Darraj *et al* ‎included a well-distributed sample across all age groups. Their results demonstrated that poor ‎sleep quality tends to worsen with age, highlighting why balanced sampling is essential[6]. Under-representing younger patients introduces a clear bias because age is a strong determinant of ‎both diabetes complications and sleep quality. Ignoring this limitation can weaken the study’s ‎findings, thus making it less convincing and invalidating its outcomes.‎

In conclusion, although the study offers valuable insights, some ‎‎‎‎‎aspects ‎‎need to be addressed to increase the reliability ‎of the results. Conducting this study by analyzing sleep determinants, better interpretation, a ‎multivariate regression model, and balanced age sampling would ‎‎significantly ‎‎increase the reliability of the study.‎

## Data Availability

Not applicable.
